# The Supply of Doctors

**Published:** 1920-06-12

**Authors:** 


THE PUBLIC WELL-BEING—(*continued*)

### The Supply of Doctors.

Sir Donald MacAlister, the president of the General Council of Medical Education and Registration of the United Kingdom, gave some interesting figures and comments on the supply of doctors—men and women—at the Council's summer session. He said that in the new Medical Register only 872 practitioners were registered in the home list, but 450 were registered in the colonial and foreign lists. The result is, the total number of new names is higher than in any year since 1915. The proportion of women practitioners is increasing and is likely to increase rapidly during the next year or two. He learns, however, on good authority, that their services are in less demand than during

**THE PUBLIC WELL-BEING—(*continued*).**

the war, and that newly-qualified women are finding difficulty in obtaining suitable opportunities for professional work. Supply and demand will no doubt adjust themselves in time; but in view of the large entry of women students it is proper to warn those concerned that, in the meantime, individual disappointments may be encountered. The profession of medicine is, in regard to women, affected, like other occupations, by the return to civil life of large numbers of ex-Service men as students and practitioners. To those men the country owes special consideration, and they ought to receive it. But inevitably they tend to displace a proportion of the women who so capably carried on the work of the profession during their absence abroad.

The "Medical Student's Register" indicates that the depletion of their professional ranks by the wastage of the war will in a few years be much more than made good, for no fewer than 3,420 medical students (men and women) were registered in 1919 as compared with 1,600 in 1914. The number of registrations, indeed, exceeds by over 1,000 the highest previously recorded—namely, in 1891—when for special reasons the number rose to 2,405. This sudden increase cannot at once be met by a corresponding expansion of their educational resources, and the strain thrown upon the medical schools of the country is therefore for the time excessive. In the departments of anatomy and operative surgery, of midwifery, and of clinical medicine, the existing provision is no longer fully adequate to the new demands.

